# Swiss Validation of the Enhanced Recovery After Surgery (ERAS) Database

**DOI:** 10.1007/s00268-020-05926-z

**Published:** 2021-01-23

**Authors:** Basile Pache, David Martin, Valérie Addor, Nicolas Demartines, Martin Hübner

**Affiliations:** 1 Department of Visceral Surgery, Lausanne University Hospital CHUV, Bugnon 46, 1011 Lausanne, Switzerland; 2 Department of Gynecology, Lausanne University Hospital CHUV, Pierre Decker 2, University of Lausanne (UNIL), Lausanne, 1011 Switzerland

## Abstract

**Background:**

Enhanced recovery after surgery (ERAS) pathways have considerably improved postoperative outcomes and are in use for various types of surgery. The prospective audit system (EIAS) could be a powerful tool for large-scale outcome research but its database has not been validated yet.

**Methods:**

Swiss ERAS centers were invited to contribute to the validation of the Swiss chapter for colorectal surgery. A monitoring team performed on-site visits by the use of a standardized checklist. Validation criteria were (I) coverage (No. of operated patients within ERAS protocol; target threshold for validation: ≥ 80%), (II) missing data (8 predefined variables; target ≤ 10%), and (III) accuracy (2 predefined variables, target ≥ 80%). These criteria were assessed by comparing EIAS entries with the medical charts of a random sample of patients per center (range 15–20).

**Results:**

Out of 18 Swiss ERAS centers, 15 agreed to have onsite monitoring but 13 granted access to the final dataset. ERAS coverage was available in only 7 centers and varied between 76 and 100%. Overall missing data rate was 5.7% and concerned mainly the variables “urinary catheter removal” (16.4%) and “mobilization on day 1” (16%). Accuracy for the length of hospital stay and complications was overall 84.6%. Overall, 5 over 13 centers failed in the validation process for one or several criteria.

**Conclusion:**

EIAS was validated in most Swiss ERAS centers. Potential patient selection and missing data remain sources of bias in non-validated centers. Therefore, simplified validation of other centers appears to be mandatory before large-scale use of the EIAS dataset.

**Supplementary Information:**

The online version contains supplementary material available at (10.1007/s00268-020-05926-z).

## Introduction

Enhanced recovery after surgery (ERAS) pathways have largely contributed over the last two decades to optimize perioperative care for numerous surgical procedures and to improve postoperative outcomes [1]. ERAS guidelines are valuable tools to facilitate safe implementation and high degree of standardization among centers. However, the underlying evidence for most perioperative care items is weak or modest, frequently issued by indirectness from similar surgical procedures [2, 3]. Therefore, prospective monitoring of feasibility and outcomes is key component of ERAS philosophy. The ERAS interactive audit system (EIAS) is an interactive online platform helping ERAS centers to carry out auditing of their performance. In addition, EIAS could be a powerful tool to perform outcome research on a larger scale with currently about 60,000 patient entries only for colorectal surgery worldwide. Quality of the entered data has not been tested so far, neither on national or international level.

The aim of this study was to validate the Swiss EIAS dataset for colorectal surgery.

## Materials and methods

### Participants and study design

This prospective validation study aimed to monitor data sets for colorectal surgery procedures in all ERAS centers in Switzerland. For this purpose, center leaders received repeated written personal invitations and, if needed, additional phone calls (over/up to three times). Centers that did not respond or whose data were not accessible were excluded. The study focused on patients with colorectal and small bowel resections, but excluded those requiring emergency surgery and patients unwilling to consent to inclusion in the register.

### EIAS validation process

Onsite visits were performed by two members of the expert center (BP, VA) which analyzed three criteria by use of a standardized checklist (online appendix 1). Criteria and target thresholds had been defined in 2017 in Lyon (FR) by the Swedish and Swiss ERAS teams.

#### Coverage

Completeness of ERAS care and assessment of potential patient selection was checked by comparing the list of consecutive eligible colorectal ERAS patients from hospital's administrative records with patients that had entered the EIAS during a one-year period of time. A maximal threshold of excluded patients was set at < 20%.

#### Missing data

Eight key variables were selected to check the completeness of entries in EIAS: preoperative bowel preparation, preoperative carbohydrate drink, intravenous fluids on day 0 (volumes infused per-operatively, in recovery room and in ward), withdrawal of urinary catheter on day 1, mobilization on day 1, any complications, length of stay, and reoperation. A maximal threshold of missing data was set at ≤ 10% for validation.

#### Accuracy of data

Consistency of EIAS entries for hospital length of stay and complications (the most severe, according to Clavien-Dindo [4]). A maximal threshold for inaccurate recording of these key outcome measures was set at 20% for validation.

These three criteria were assessed, through on-site visit, under direct supervision from the local host, by comparing EIAS entries with the medical charts of a random sample of patients per center (range 15–20 patients, depending on the year of EIAS implementations and number of patients available). Validation visits were performed between April 2018 and December 2019. All centers were audited for the same year (2017). If 1 out of the 3 analyzed criteria were not fulfilled for a center, it meant a failure for the validation process. Exploratory interviews were conducted among the local ERAS teams inquiring about difficulties with data entry and key of success for complete and high-quality entries.

Formal approval was waived by the institutional review board as no individual patient information was retained for the reporting of study results. Patient informations were handled by the local team, with no direct access to data by the visiting audit experts.

### Statistical analysis

Descriptive statistics were employed for analysis of results and reported as number and percentage. Coverage was calculated as the number of EIAS-listed patients divided by the number of eligible patients. Missing data was calculated by the number of missing variables for all analyzed patients per center divided by the number of assessed variables (number of patients × 8). Accuracy was computed by dividing the total of correct entries per center for complications and hospital stay by the number of patients (× 2).

## Results

**Fig. 1:**
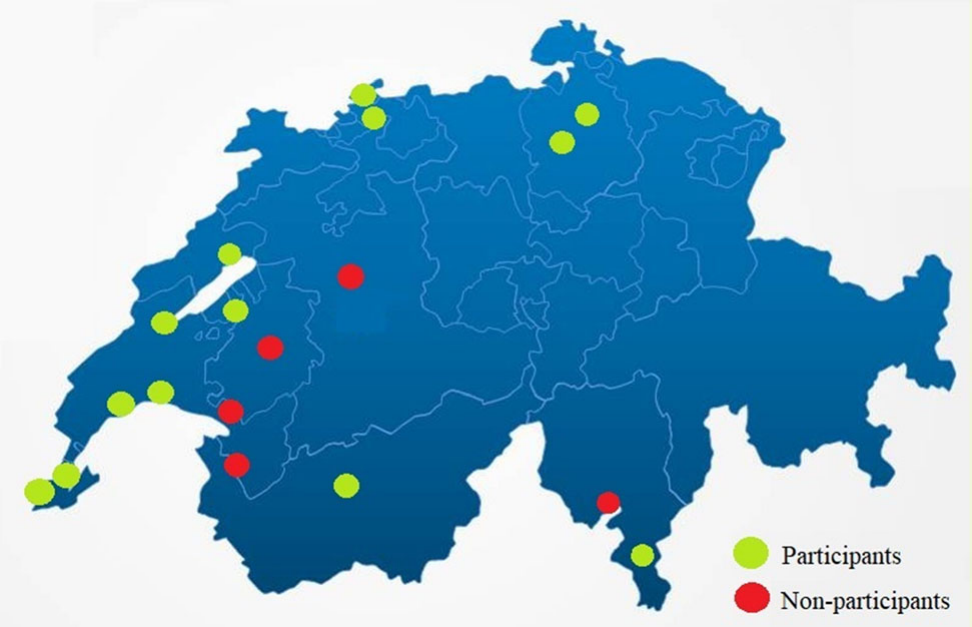
Swiss colorectal ERAS centers. Geographic distribution of Swiss colorectal ERAS centers. Green color indicates participation in the validation process

Out of a total of 18 Swiss colorectal ERAS centers, 1 center refused participation, 2 others did not respond and 2 centers had to be excluded due to the impossibility of data access during the visit. Thirteen centers (72%) could be included in the validation process as depicted in Fig. 1.

The overall results of the validation process are presented in Table 1. ERAS coverage was available in only 7 centers (54%) and varied between 76 and 100%. Overall missing data rate was 5.7% and accuracy for length of hospital stay and complications was overall 84.6%. EIAS data sets were validated for 8 out of 13 centers (62%), while 5 centers (38%) failed in the validation process for one or several reasons as detailed in Table 1. Details of missing data and accuracy are presented in online appendix 2.

**Table 1:** Coverage, missing data and accuracy of entries in the Swiss colorectal ERAS cohort

Center	Coverage	Missing	Accuracy	Validation
1	196/233 (84.1%)	2/160 (1.25%)	36/40 (90%)	Yes
2	77/86 (89.5%)	0/160 (0%)	35/40 (87.5%)	Yes
3	NA	0/120 (0%)	30/30 (100%)	Yes
4	NA	7/160 (4.4%)	32/40 (80%)	Yes
5	**73/95 (76.8%)**	**21/160 (13.1%)**	38/40 (95%)	**No**
6	67/77 (87.0%)	12/160 (7.5%)	**29/40 (72.5%)**	**No**
7	NA	7/160 (4.4%)	36/40 (90%)	Yes
8	NA	3/160 (1.9%)	24/30 (80%)	Yes
9	**131/171 (76.6%)**	14/160 (8.8%)	**27/40 (67.5%)**	**No**
10	129/130 (99.2%)	16/160 (10%)	**29/40 (72.5%)**	**No**
11	NA	8/160 (6%)	32/40 (80%)	Yes
12	166/166 (100%)	**21/160 (13.1%)**	36/40 (90%)	**No**
13	NA	5/160 (3.1%)	38/40 (95%)	Yes
Total	Range: 76–100%	Mean 5.7% (SD 4.4)	Mean: 84.6% (SD 9.60)	8/13

The validation criteria and minimal requirements for validation were as follows:

(I) Coverage: No. of operated patients within ERAS protocol/total No. of operated patients); ≥ 80%

(II) Missing data for 8 variables: missing/total; ≤ 10%

(III) Accuracy for 2 variables: accurate/total; ≥ 80%

The denominators for II and III depend on the number of patients in the random sample. Values not meeting the minimal thresholds are indicated in **bold**. One criterion failed resulted in non-validation

Totals for all centers are range for coverage, means for missing data and accuracy and sum for validated centers

Exploratory interviews helped to identify several key points and problems related to efficient, complete and accurate data entry. These were mainly “maintain interest of the nursing and medical teams on ERAS pathways and data filing of the patient’s ERAS booklet,” “sufficient supervision and support from team leaders for cross-checking data and maintaining the team cohesion with positive feedback,” “sufficient dedicated time for collecting data and EIAS database filling,” “little or no recognition from institutional direction to maintain the ERAS program.”

## Discussion

This study is the first validation of a subset of the EIAS, one of the largest perioperative databases worldwide. This monitoring process showed overall satisfactory data quality with the validation of a majority of centers. However, the results of this study clearly put question marks for uncritical use of EIAS data from non-monitored or non-validated centers, with regards to potential patient selection and missing data.

Large-volume databases are frequently used in surgical research. National databases have been used to develop risk stratification tools, assess postoperative complications, calculate costs and investigate other factors across multiple surgical specialties [5, 6]. The results from these databases provide better evidence-based guidelines for decisions made regarding patient care preoperatively, intraoperatively, and postoperatively [7, 8]. With regard to patient selection, differing strategies across databases can inevitably contribute to differing results for similar patient cohorts, and they may define certain complications or comorbidities differently [5]. A meta-analysis showed that clinical studies that used observational databases could be sensitive to the choice of database, and that study results may shift from statistical significance from one extreme to the other [9]. For these many reasons, EIAS validation was required in order to confirm it as a potential research tool for quality control and surgical research. The results of the present study showed that some data were uncorrectly reported, at least in the Swiss setting. In comparison, many studies have been published using data from unsatisfactory maintained database, leading to withdrawal or retraction of publications [10]. The National Surgical Quality Improvement Program (NSQIP) is an ongoing quality management initiative that applies the methodology developed and validated to all the Veterans Affair Medical Centers (VAMCs) that perform major surgery [1, 2]. More than 130 clinical variables are collected for each case by trained surgical clinical reviewers (SCR), who are rigorously trained and audited to ensure data reliability [3]. The inter-rater reliability (IRR) audit has been used to assess the quality of the NSQIP data collected [3]. This process involved the review of 12 to 15 charts per institution and time period audited. Charts were selected based on criteria designed to identify potential reporting errors, such as cases with five or more preoperative risk factors and no reported mortality or morbidity, or cases with two or fewer preoperative risk factors and reported mortality or morbidity. The site visitor reviewed more than 100 variables for each case. The disagreement rate between the SCR and the site reviewer was calculated as a percentage using the number of disagreements divided by the total number of variables reviewed. One study using this methodology showed that the overall disagreement rate was 1.56% in 2008, and estimated kappa values suggested substantial or almost perfect agreement for most variables [3]. Another study assessed readmission data captured in NSQIP at a single academic institution and compared it with data abstracted from the medical record and administrative data [4]. Of 1748 patient entries, NSQIP had very high agreement with chart review for identifying all-cause readmission events (κ = 0.98). Interestingly, agreement with chart review on the cause of readmission was higher for NSQIP (κ = 0.75) than for administrative data (κ = 0.46). Repetitive monitoring and validation are therefore needed. However, there is currently no standardized methodology for large database validation. In the present study, the validation criteria were established as objectively as possible and before carrying out the visits to the centers. It is difficult to compare and judge the quality of similar efforts, but a less stringent approach would have probably brought “better” results.

In the present study, validation methodology was discussed between several stakeholders, not only in Switzerland, but also with the EIAS management team in Sweden, in order to achieve the highest standard for data quality. Validation criteria have been set at ≥ 80% percent agreement for coverage and accuracy, and ≤ 10% missing data was tolerated. When validating key variables from EIAS, all medical records were retrieved enabling the complete validation of selected variables, as performed previously for a Danish urogynaecological database validation [11]. Similar to the present results, the overall percent agreement between selected variables and medical records was at least 90%. Selected variables were of major importance in relation to surgical procedures performed, but possible selection bias may have occurred. Other factors such as precision, consistency and timeliness have also been used in database validation [12, 13] However, there is currently no clear consensus on the criteria for validating a surgical database.

Unfortunately, among the visited centers in this study, coverage could not be assessed in 6 centers out of 13. The main reasons evoked by centers were the difficulty to extract the correct colorectal interventions among other surgeries performed within their hospitals. Potential explanations include a complex way of coding in Switzerland, and varying IT administrative support in the visited hospitals. However, the deliberate patient selection seemed unlikely, and the reasons for this low rate were rather due to these opaque and complicated administrative and coding issues. Therefore, centers without available coverage were not automatically excluded from the further validation process. It is essential to think of solutions for the future of this kind of research. Centers with an episode of bad coverage could remain included in analyses but should be advertised to take measures to remediate this crucial point. Repeated violation should result in exclusion from further analyses as deliberate patient selection could not be excluded anymore. These important rules should be discussed and decided by the scientific and executive committee of societies leading large databases.

Missing data rate was 5.7% and accuracy was 84.6%. These missing or wrongly labeled data might be due to insufficient supervision and training of ERAS study nurses and data managers, as well as poorly filled ERAS patient’s booklet. As shown in a qualitative study, barriers to ERAS implementation are multiple and include in particular time restraints, opposing colleagues and logistical reasons [14, 15]. Data entry could also be influenced by time scale, with a better quality of data for centers who were recently implemented, as it was reported that compliance decreases with time [2]. The results of this present study should therefore be interpreted with caution, and it cannot be extrapolated to the entire Swiss colorectal or even global EIAS dataset. It should also be remembered that missing data are difficult to interpret in a validation process and that it all comes down to the importance of variables that are measured. Some of them, such as complications, seem more important and should maybe be weighted differently than others (mobilization after surgery for example). This was not done in the current study, and the results may have changed. However, validation criteria for missing data were elaborated by the Swiss-Swedish core teams based on a selection of key and varied objective criteria among all the ERAS items, in particular 2 preoperative items, 1 intraoperative item, 2 postoperative items and 2 outcomes, namely length of stay and complications. Furthermore, the importance of complication and length of stay data was, however, assessed by accuracy. Thus, an overview was presented in this study, and the rate of missing values was ultimately low and accuracy was high. Future efforts should concentrate on the definition of valid proxies to allow for reliable and easy-to-perform validation of the entire dataset.

In Switzerland, CHOP (Swiss Classification of Surgical Interventions) codes could be used to standardize the extraction of interventions from the institutional surgical program for validation auditing. A common limitation is that these codes were not originally developed for research purposes and their use may only be valid for certain diagnoses, procedures or complications. Furthermore, extraction of these CHOP codes often comes from insurance claims or hospital-level records, which may be influenced by reimbursement strategies or coded by non-medical team members [5].

Exploratory interviews with the local ERAS teams about difficulties with data entry and key of success for complete and high-quality entries showed the reality of fieldwork. Most of the nurses showed great interest in their job but somehow felt left to their own devices. There should be ways to make their job easier, for instance with easy-to-use automated extraction from the electronic patient record directly to the EIAS database. This would enable spare time to cross-check data, and spend time with nursing and medical team in the day-to-day clinic, to teach and motivate them to fulfill ERAS items. A good practical example of the crucial role of the ERAS nurse is that during this study, several patients not included in EIAS or included but with most missing value, were those during the holidays of the ERAS nurse.

Multidisciplinary teamwork, with continual internal audit and meeting on a regular basis, is key to success in maintaining higher compliance to ERAS guidelines [16]. ERAS nurse is a key component, as facilitator and cornerstone of the whole surgical team, as a bridge between patients reality, ward nurses and medical staff [17]. Data feed back to local team is important in order to maintain high motivation and adherence to ERAS protocols [15].

There are several limitations to this study that need to be discussed. The validation criteria were developed after expert consensus, but in an arbitrary manner, insofar as there are no clearly validated criteria in today’s literature. Only 13 out of 18 centers (72%) in Switzerland were included in the study, introducing a potential selection bias. The data of the centers that were not visited were unknown. This aspect might have influenced the final results, thus limiting the generalization of validation in Switzerland and worldwide. It can be suspected, however, that centers participating in the validation process were more motivated in good ERAS outcomes than those who declined. Thus, the participating centers may present the best results in daily clinical practice and be representative of the good ERAS program. The present validation process should be extended to other countries and then data compared, in order to validate or offer opportunities to improve EIAS.

In conclusion, EIAS was validated in most Swiss ERAS centers, but issues of concern were raised for coverage and missing data in particular. Therefore, an improved and easier process shall be elaborated in order to facilitate validation of the entire dataset before its large-scale use for outcome research.

## Supplementary Information

Supplementary file1 (DOCX 49 kb)

Supplementary file2 (DOCX 46 kb)
