# Supplementary material for: Swiss Validation of the Enhanced Recovery After Surgery (ERAS) Database
Source: World J Surg. 2021 Jan 23;45(4):940–5. doi: 10.1007/s00268-020-05926-z (PMC7921022; doi:10.1007/s00268-020-05926-z)
Supplement: Supplementary file 1 — Supplementary file1 (DOCX 49 kb) [file 268_2020_5926_MOESM1_ESM.docx]

**Online appendix 1** EIAS validation checklist

1. **Coverage:**

| Number of registered patients in the ERAS database  (12 months) | Number of operated patients NOT registered in the ERAS database (12 months) |
| --- | --- |
|  |  |

1. **Missing values**:

| **Patient n°** | **EIAS ID n°** | **Bowel preparation** | **Preoperative drink** | **Intravenous fluids day 0** | **Withdrawal of urinary catheter day 1** | **Mobilisation on day 1** | **Any complications** | **Length of stay** | **Reoperation** |
| --- | --- | --- | --- | --- | --- | --- | --- | --- | --- |
| **1** |  |  |  |  |  |  |  |  |  |
| **2** |  |  |  |  |  |  |  |  |  |
| **3** |  |  |  |  |  |  |  |  |  |
| **4** |  |  |  |  |  |  |  |  |  |
| **5** |  |  |  |  |  |  |  |  |  |
| **6** |  |  |  |  |  |  |  |  |  |
| **7** |  |  |  |  |  |  |  |  |  |
| **8** |  |  |  |  |  |  |  |  |  |
| **9** |  |  |  |  |  |  |  |  |  |
| **10** |  |  |  |  |  |  |  |  |  |
| **11** |  |  |  |  |  |  |  |  |  |
| **12** |  |  |  |  |  |  |  |  |  |
| **13** |  |  |  |  |  |  |  |  |  |
| **14** |  |  |  |  |  |  |  |  |  |
| **15** |  |  |  |  |  |  |  |  |  |
| **16** |  |  |  |  |  |  |  |  |  |
| **17** |  |  |  |  |  |  |  |  |  |
| **18** |  |  |  |  |  |  |  |  |  |
| **19** |  |  |  |  |  |  |  |  |  |
| **20** |  |  |  |  |  |  |  |  |  |

1. **Accuracy:**

| **Patient n°** | **EIAS ID n°** | **Length of stay** | | **Number of complications** | |
| --- | --- | --- | --- | --- | --- |
|  |  | **EIAS database** | **Patient medical record** | **EIAS database** | **Patient medical record** |
| **1** |  |  |  |  |  |
| **2** |  |  |  |  |  |
| **3** |  |  |  |  |  |
| **4** |  |  |  |  |  |
| **5** |  |  |  |  |  |
| **6** |  |  |  |  |  |
| **7** |  |  |  |  |  |
| **8** |  |  |  |  |  |
| **9** |  |  |  |  |  |
| **10** |  |  |  |  |  |
| **11** |  |  |  |  |  |
| **12** |  |  |  |  |  |
| **13** |  |  |  |  |  |
| **14** |  |  |  |  |  |
| **15** |  |  |  |  |  |
| **16** |  |  |  |  |  |
| **17** |  |  |  |  |  |
| **18** |  |  |  |  |  |
| **19** |  |  |  |  |  |
| **20** |  |  |  |  |  |
