# Supplementary material for: Swiss Validation of the Enhanced Recovery After Surgery (ERAS) Database
Source: World J Surg. 2021 Jan 23;45(4):940–5. doi: 10.1007/s00268-020-05926-z (PMC7921022; doi:10.1007/s00268-020-05926-z)
Supplement: Supplementary file 2 — Supplementary file2 (DOCX 46 kb) [file 268_2020_5926_MOESM2_ESM.docx]

**Online appendix 2** EIAS validation checklist

| **Center** | **Missing data** | | | | | | | | **Accuracy** | | | |
| --- | --- | --- | --- | --- | --- | --- | --- | --- | --- | --- | --- | --- |
|  | Bowel preparation | Preoperative drink | Intravenous fluids day 0 | Withdrawal of urinary catheter day 1 | Mobilisation on day 1 | Any complications | Length of stay | Reoperation | Accumulated length of stay (days) | | Accumulated complications (number) | |
|  |  |  |  |  |  |  |  |  | EIAS | chart | EIAS | chart |
| 1 | 20/20 | 20/20 | 20/20 | 20/20 | 18/20 | 20/20 | 20/20 | 20/20 | 197 | 197 | 22 | 27 |
| 2 | 20/20 | 20/20 | 20/20 | 20/20 | 20/20 | 20/20 | 20/20 | 20/20 | 161 | 161 | 8 | 8 |
| 3 | 15/15 | 15/15 | 15/15 | 15/15 | 15/15 | 15/15 | 15/15 | 15/15 | 80 | 80 | 3 | 3 |
| 4 | 20/20 | 19/20 | 19/20 | 17/20 | 18/20 | 20/20 | 20/20 | 20/20 | 202 | 208 | 24 | 26 |
| 5 | 20/20 | 19/20 | 15/20 | 19/20 | 6/20 | 20/20 | 20/20 | 20/20 | 203 | 203 | 9 | 9 |
| 6 | 20/20 | 19/20 | 20/20 | 11/20 | 18/20 | 20/20 | 20/20 | 20/20 | 193 | 195 | 9 | 13 |
| 7 | 20/20 | 20/20 | 17/20 | 18/20 | 18/20 | 20/20 | 20/20 | 20/20 | 146 | 146 | 7 | 10 |
| 8 | 15/15 | 15/15 | 14/15 | 13/15 | 15/15 | 15/15 | 15/15 | 15/15 | 89 | 90 | 18 | 18 |
| 9 | 20/20 | 20/20 | 20/20 | 10/20 | 16/20 | 20/20 | 20/20 | 20/20 | 309 | 324 | 26 | 33 |
| 10 | 20/20 | 20/20 | 16/20 | 14/20 | 14/20 | 20/20 | 20/20 | 20/20 | 226 | 225 | 17 | 26 |
| 11 | 20/20 | 20/20 | 18/20 | 14/20 | 20/20 | 20/20 | 20/20 | 20/20 | 167 | 167 | 6 | 10 |
| 12 | 20/20 | 20/20 | 15/20 | 17/20 | 17/20 | 20/20 | 20/20 | 20/20 | 141 | 143 | 6 | 3 |
| 13 | 20/20 | 20/20 | 20/20 | 20/20 | 15/20 | 20/20 | 20/20 | 20/20 | 155 | 155 | 15 | 14 |
| **Total** | 250/250 | 247/250 | 229/250 | 209/250 | 210/250 | 250/250 | 250/250 | 250/250 | 2269 | 2294 | 170 | 200 |
| **Percent agreement** | 100% | 98.8% | 91.6% | 83.6% | 84% | 100% | 100% | 100% | 98.9% | | 85% | |
